# The exponential increase in the number of prescriptions for ADHD medication between 2012 and 2022 in Poland in patients aged 5–59 years: a pharmacoepidemiological study

**DOI:** 10.1007/s00787-026-03104-5

**Published:** 2026-06-19

**Authors:** Marcin Rzeszutek, Łukasz Konowałek, Joanna Martin, Agnieszka Butwicka, Tomasz Wolańczyk

**Affiliations:** 1 Department of Child Psychiatry, Medical University of Warsaw, Żwirki i Wigury 63A, 02-091 Warsaw, Poland; 2 Department of Medical Epidemiology and Biostatistics, Karolinska Institutet, Stockholm, Sweden; 3 Division of Mental Health Services, R&D Department, Akershus University Hospital, Lørenskog, Norway; 4 Institute of Clinical Medicine, Faculty of Medicine, University of Oslo, Oslo, Norway; 5 Department of Biostatistics and Translational Medicine, Medical University of Lodz, Lodz, Poland; 6 Faculty of Medicine, Uppsala University, Uppsala, Sweden; 7 Centre for Neuropsychiatric Genetics and Genomics, Cardiff University, Cardiff, UK; 8 Wolfson Centre for Young People’s Mental Health, Cardiff University, Cardiff, UK

**Keywords:** Methylphenidate, Atomoxetine, Drug utilization, Drug prescriptions, Time factors

## Abstract

There are currently no pharmacoepidemiological data on the use of ADHD medications in Poland. Moreover, evidence from Central and Eastern Europe remains limited, despite a global increase in ADHD diagnosis and pharmacological treatment. This lack of evidence highlights the need for population-based analyses of prescribing trends in this setting. This study aims to analyze data from the Polish prescription register to estimate the increase in the total number of prescriptions for methylphenidate and atomoxetine from 2012 to 2022, as well as identify any factors that may have influenced this trend. A retrospective population-based study utilizing a large electronic database containing all (*n* = 925 536) prescriptions for ADHD medication in Poland for the period between 2012 and 2022 for patients aged 5–59 years was conducted. We performed regression-based temporal trend analyses, including age- and sex-stratified interaction models. The prevalence of ADHD medications rose from 170.33 prescriptions per 100 000 in 2012 (*n* = 48,429) to 591.50 per 100 000 in 2022 (*n* = 155,068), marking a 247% increase. Methylphenidate remained the most prescribed medication, with its prevalence increasing from 168.28 prescriptions per 100,000 in 2012 to 481.45 prescriptions per 100,000 in 2022 (186% increase), while atomoxetine experienced an increase from 2.02 prescriptions per 100,000 in 2012 to 110.05 prescriptions per 100,000 in 2022 (5242% increase). ADHD medication usage increased across all age groups, with the highest prescription rates observed among children aged 7–12 and significant growth seen in adolescents aged 13–17. The use of ADHD medications has increased among all age groups and in males as well as females. The rate of increase was highest among females, particularly in the group of young adults (18–24 year olds). Our study provides data on prescription trends in Poland, contributing to the limited evidence from Central and Eastern Europe.

## Introduction

Attention deficit hyperactivity disorder (ADHD) is a neurodevelopmental disorder manifested by a persistent pattern of inattention and/or hyperactive and impulsive behavior [1]. Available guidelines recommend non-pharmacological and pharmacological treatment to reduce the severity of symptoms and improve patient’s functioning. Pharmacological first-line treatment includes psychostimulants such as methylphenidate and amphetamines. Second-line treatments include non-stimulants such as atomoxetine, clonidine, and guanfacine [2].

A recent systematic review of worldwide ADHD prevalence highlights substantial heterogeneity depending on study methodology. Prevalence estimates vary from approximately 1.6% in register-based studies to 4.2–4.8% in clinical studies and up to 5.0% in survey-based studies, reflecting differences in diagnostic approaches and data sources [3].

In Poland ADHD diagnoses are conducted both in public clinics and private healthcare facilities. However, diagnostic data from private healthcare providers are not consistently captured in a single and fully comprehensive national registry. According to governmental sources, the prevalence of ADHD in Poland in 2022 was estimated at 0.66%. This figure was calculated based on a database that relies on ADHD diagnoses made according to the ICD-10 classification and includes information on the number of living patients for whom a healthcare service with the primary diagnosis code F90 was reported in the public healthcare data in 2022 or earlier. Unfortunately, no data is available regarding the number of individuals receiving treatment through private healthcare services. We expect that ADHD prevalence rates in Poland should be similar to those observed in other parts of the world. Therefore, it is reasonable to assume that the available data may be underestimated.

ADHD medication prescribing trends are increasing in all age groups in North America and both Northern and Western Europe. In a retrospective study, using population data from 13 countries, the absolute increase in ADHD medication use prevalence per year ranged from 0.02% to 0.26% and from 0.0006% to 0.12% for children and adults respectively [4]. Another longitudinal study involving 64 countries, including Poland, showed that multinational ADHD medication consumption increased by 9.72% (95% confidence interval [CI], 6.25%, 13.31%) per year between 2015 and 2019 [5]. The growth in ADHD medication usage, observed in recent decades is greater for females than males in Western/European countries [6]. It remains, however, unknown whether this phenomenon is specific to Western European countries.

Up-to-date data on ADHD medication prescribing trends in Eastern European countries, including Poland, remain limited in comparison with Western and Scandinavian countries. With its population of over 38 million and its significant economic growth since the fall of the Eastern Bloc, Poland presents a unique opportunity to examine the trends and patterns of ADHD medication prescriptions in this region. Therefore, this nationwide registry-based descriptive study aims to analyze data from the Polish Prescription Register to estimate the increase in the total number of prescriptions for methylphenidate and atomoxetine from 2012 to 2022, as well as to identify demographic factors that may have influenced this trend, such as age and sex.

## Materials and methods

### Data sources

Prescription data for the period between 2012 and 2022 were obtained from the Nationwide Prescription Register (e-Health Centre), a nationwide administrative database covering all prescribed medications in Poland, which supports the completeness and reliability of the data for pharmacoepidemiological research. The Nationwide Prescription Register has been previously used in published studies [7, 8]. The data was provided by the Department of Public Health of the Ministry of Health of the Republic of Poland. In Poland, the only psychostimulant available during the study period was methylphenidate, offered in immediate-release, controlled-release, and OROS (osmotic-controlled release oral delivery system) formulations. The only officially approved non-psychostimulant for the treatment of ADHD in Poland was atomoxetine. Methylphenidate and atomoxetine are both approved for use in children and adolescents older than 6 years old and adults.

The data contained the number of prescriptions, the year of prescription, age, sex and the anatomical therapeutic chemical (ATC) code.

### Study population

We have included the data on prescriptions for Polish residents between 5 and 59 years of age (*n* = 925,536 prescriptions).

Prescription data were stratified and divided into subgroups according to age. The age groups for children and adolescents were established based on the developmental stages: preschoolers (5–6 years), children (7–12 years), and adolescents (13–17 years). To highlight age-dependent prescription trends in adults, they were divided into 6 groups with 6-year intervals. We calculated the annual prevalence for both medications in the total population and for males and females separately and a relative change for each age group and sex. To calculate the prevalence rates, we used the number of prescriptions per 100,000 individuals, with the total number of individuals in the relevant age group and sex in Poland per year as the denominator. To emphasize the differences in the dynamics of changes between individual age groups and sex, the relative change in prevalence between 2012 and 2022 was calculated using the following formula:

$$\:Relative\:change\:=\:\frac{prevalence\:in\:2022-prevalence\:in\:2012}{prevalence\:in\:2012}$$

### Statistical analysis

We first assessed whether temporal changes in ADHD medication prescription counts were better characterized by a linear or exponential pattern. This was evaluated by comparing regression models using untransformed and log-transformed prescription counts with likelihood ratio tests.

To examine whether temporal changes in ADHD medication prescribing differed across demographic groups, we conducted an age- and sex-stratified trend analysis. Prescription counts were aggregated by calendar year, medication type, sex, and age group. Age groups were defined as 5–6, 7–12, 13–17, 18–24, 25–31, 32–38, 39–45, 46–52, and 53–59 years, consistent with the descriptive analyses.

We fitted negative binomial regression models for prescription counts, separately for any ADHD medication, methylphenidate, and atomoxetine. Negative binomial models were used to account for overdispersion in count data. Calendar year was modelled as a continuous variable, centered at 2012. To assess whether temporal trends differed by demographic group, interaction terms between calendar year and sex and between calendar year and age group were included. The 7-12-year age group and males were used as reference categories. Models with interaction terms were compared with models including only main effects using likelihood ratio tests. Results are presented as interaction incidence rate ratios (IRRs), where values above 1 indicate a steeper annual increase in prescription counts compared with the reference group.

These interaction models were fitted to prescription counts and therefore evaluate heterogeneity in prescription count trends rather than differences in the number of treated individuals.

## Results

The number of prescriptions issued annually ranged between 48,429 in 2012 and 155,068 in 2022. Although the proportion of prescriptions issued for males was higher than that for females across all years, the proportion of prescriptions for females increased gradually over time. The highest percentage of prescriptions in all the years covered by the study was for the age group 7–12. The increase in the prescription count was most pronounced within the age group 13–17. Table 1 presents the absolute values and the percentage values of the number of prescriptions for the following years, categorized by sex and age group.

**Table 1:** Demographic characteristics of the study population receiving any ADHD medication prescription from 2012–2022

Characteristics	2012	2013	2014	2015	2016	2017	2018	2019	2020	2021	2022
Number of prescriptions (n)	48429	56442	66294	63015	68310	74290	80413	87143	107691	118441	155068
Sex (n)											
Male	42762 (88.30%)	49725 (88.10%)	58352 (88.02%)	55207 (87.61%)	59491 (87.09%)	64372 (86.65%)	69027 (85.84%)	74220 (85.17%)	89545 (83.15%)	96612 (81.57%)	121806 (78.55%)
Female	5667 (11.70%)	6717 (11.90%)	7942 (11.98%)	7808 (12.39%)	8819 (12.91%)	9918 (13.35%)	11386 (14.16%)	12923 (14.83%)	18146 (16.85%)	21829 (18.43%)	33262 (21.45%)
Age group, years (n)											
5–6	1093 (2.26%)	1478 (2.62%)	1925 (2.90%)	2304 (3.66%)	2772 (4.06%)	2212 (2.98%)	1821 (2.26%)	2096 (2.41%)	1233 (1.14%)	1396 (1.18%)	1812 (1.17%)
7–12	33077 (68.30%)	38615 (68.42%)	45990 (69.37%)	43737 (69.41%)	47620 (69.71%)	53044 (71.40%)	57138 (71.06%)	61293 (70.34%)	67168 (62.37%)	68536 (57.87%)	80263 (51.76%)
13–17	14002 (28.91%)	16019 (28.38%)	17974 (27.11%)	16447 (26.10%)	17182 (25.15%)	18067 (24.32%)	20230 (25.16%)	22069 (25.33%)	34661 (32.19%)	41946 (35.42%)	61812 (39.86%)
18–24	130 (0.27%)	168 (0.30%)	216 (0.33%)	232 (0.37%)	322 (0.47%)	415 (0.56%)	506 (0.63%)	697 (0.80%)	2770 (2.57%)	4037 (3.41%)	6812 (4.39%)
25–31	28 (0.06%)	47 (0.08%)	46 (0.07%)	75 (0.12%)	82 (0.12%)	100 (0.13%)	187 (0.23%)	249 (0.29%)	605 (0.56%)	1077 (0.91%)	1518 (0.98%)
32–38	49 (0.10%)	46 (0.08%)	51 (0.08%)	100 (0.16%)	148 (0.22%)	231 (0.31%)	231 (0.29%)	321 (0.37%)	460 (0.43%)	609 (0.51%)	1545 (1.00%)
39–45	25 (0.05%)	54 (0.10%)	60 (0.09%)	100 (0.16%)	149 (0.22%)	182 (0.24%)	222 (0.28%)	257 (0.29%)	462 (0.43%)	472 (0.40%)	874 (0.56%)
46–52	10 (0.02%)	4 (0.01%)	16 (0.02%)	8 (0.01%)	26 (0.04%)	20 (0.03%)	55 (0.07%)	96 (0.11%)	191 (0.18%)	193 (0.16%)	247 (0.16%)
53–59	15 (0.03%)	11 (0.02%)	16 (0.02%)	12 (0.02%)	9 (0.01%)	19 (0.03%)	23 (0.03%)	65 (0.07%)	141 (0.13%)	175 (0.15%)	185 (0.12%)

**Fig. 1:**
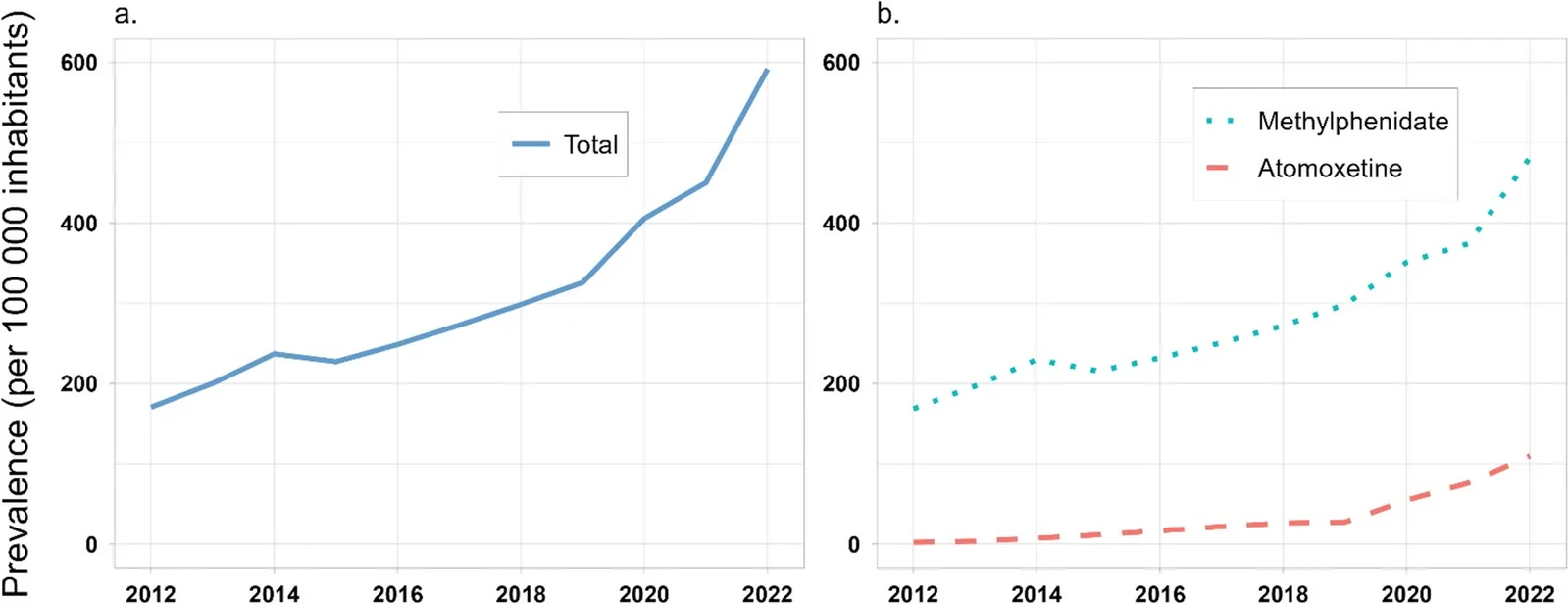
Annual prevalence of the number of prescriptions of **a** any and **b** specific medication in the years 2012–2022

The prevalence of prescriptions for both ADHD medications increased during the study period in all age groups from 170.33 prescriptions/100 000 in 2012 to 591.50 prescriptions/100 000 in 2022. Methylphenidate was the most frequently prescribed ADHD medication in all age groups and we observed its prevalence grow from 168.28 prescriptions/100 000 in 2012 to 481.45 prescriptions/100 000 in 2022. The increase in the prevalence of atomoxetine was smaller, but it progressed more rapidly from 2.02/100 000 in 2012 to 110.05/100 000 in 2022 (5242% increase) (Fig. 1.)

**Fig. 2:**
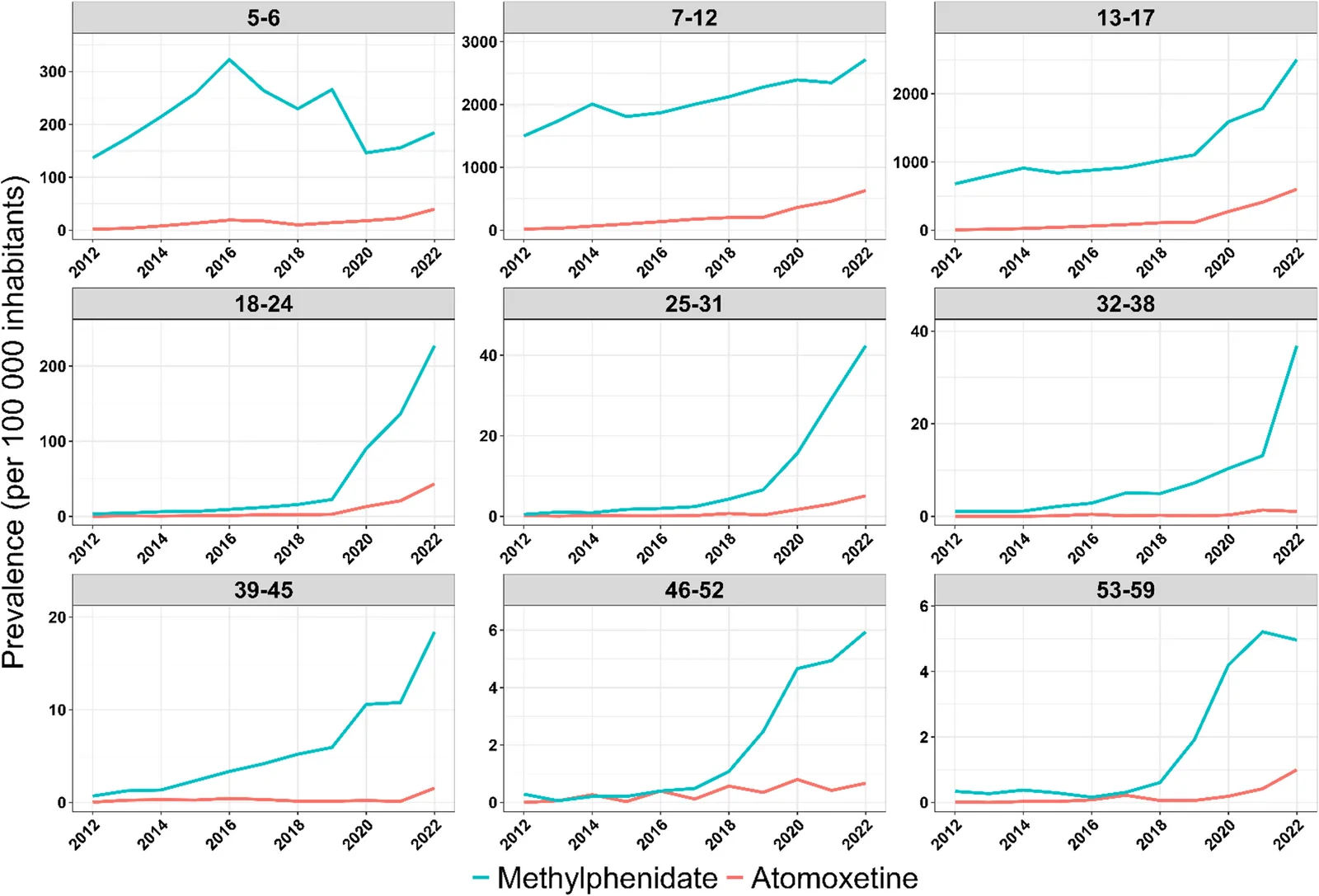
Prevalence of the number of prescriptions (per 100 000 inhabitants) for methylphenidate and atomoxetine in the years 2012–2022 by age group

The annual prevalence of both ADHD medications by age group is shown in Fig. 2. The annual prevalence increased gradually in all age groups except for preschoolers (5–6 year olds), who showed a steady rise until 2016, followed by a decline. In recent years, a gradual increase in prevalence has been observed again in this age group, with prevalence in 2022 exceeding that of 2012. The highest usage was observed for 7–12 and 13–17 year olds. The prevalence exhibited the fastest growth among young adults. Methylphenidate was most commonly used ADHD medication in all age groups and its usage increased most intensively for age group 25–31 years. The relative increase of the atomoxetine prevalence was highest among the 18–24 age group.

The prevalence of number of prescriptions for both ADHD medications stratified by sex is presented for the years 2012 and 2022 in Table 2. We observed a clear predominance of males in all age groups. The rate of increase was highest among females, particularly in the group of young adults.

**Table 2:** Prevalence of the number of prescriptions (per 100 000 males and females respectively) for all ADHD medication in 2012 and 2022 and relative change of prevalence by sex and age group

Sex	2012 prevalence	2022 prevalence	Relative change
		5–6 yrs	
M	247.55	384.60	0.55
F	23.96	54.91	1.29
		7–12 yrs	
M	2616.29	5531.00	1.11
F	360.61	1038.08	1.88
		13–17 yrs	
M	1179.11	4433.79	2.76
F	166.84	1705.94	9.23
		18–24 yrs	
M	4.95	300.47	59.70
F	2.13	238.61	111.02
		25–31 yrs	
M	0.84	59.95	70.37
F	0.41	34.60	83.39
		32–38 yrs	
M	1.56	51.27	31.87
F	0.71	24.20	33.08
		39–45 yrs	
M	1.02	28.23	26.68
F	0.41	11.55	27.17
		46–52 yrs	
M	0.29	8.89	29.65
F	0.29	4.34	13.97
		53–59 yrs	
M	0.25	8.36	32.44
F	0.47	3.66	6.79

### Exponential growth in ADHD medication prescriptions

Upon scrutinizing the temporal trends in ADHD medication prescriptions, an investigation into the growth pattern — whether linear or exponential — revealed an exponential growth pattern. The primary analysis, focused on the linear regression of calendar year on ADHD medication prescriptions, showed a notable refinement in fit when utilizing log-transformed scores.

The statistical evaluation of the likelihood ratio yielded compelling results (chi^2^ = 260.85; *p* < 0.001), underscoring a significant enhancement in model fit with the log-transformed scores. This finding supports an exponential growth pattern in characterizing the relationship between calendar year and ADHD medication prescriptions.

### Age- and sex-stratified temporal trends

The interaction model fitted the data significantly better than the main-effects model for any ADHD medication (LR = 136.76, df = 9, *p* = 4.82 × 10⁻²⁵), methylphenidate (LR = 150.32, df = 9, *p* < 0.001), and atomoxetine (LR = 41.58, df = 9, *p* < 0.001). The heterogeneity was driven mainly by age group: adult age groups, particularly 18–24 and 25–31 years, showed steeper annual increases in prescription counts than children aged 7–12 years. The calendar year-by-sex interaction was not significant for all ADHD medications combined.

**Fig. 3:**
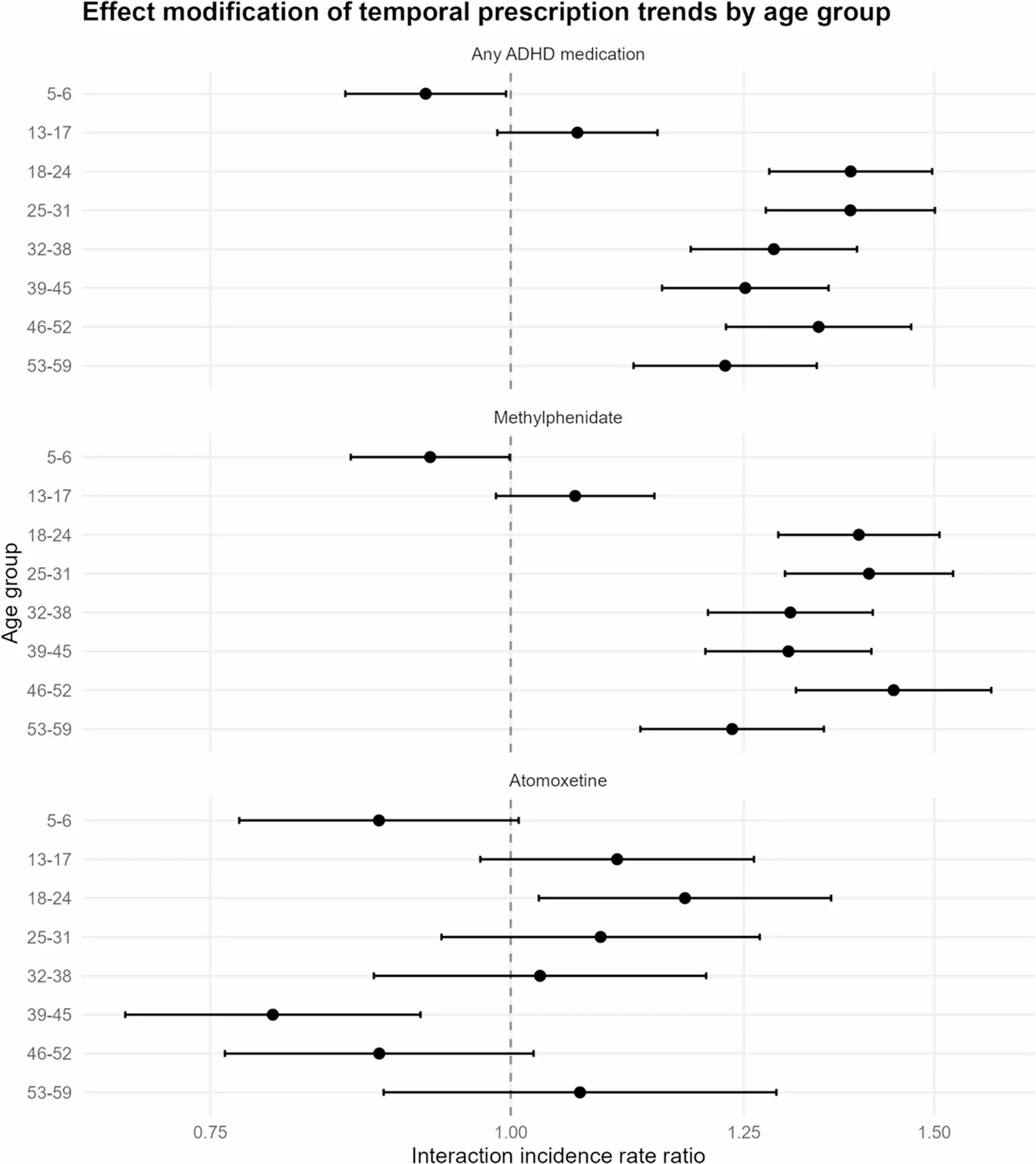
Effect modification of temporal prescription trends by age group

Figure 3 shows the age-specific interaction incidence rate ratios for temporal prescription trends, using children aged 7-12 years as the reference group. Interaction IRRs above 1 indicate age groups with steeper annual increases in prescription counts. The figure demonstrates that the increase in prescriptions was disproportionately stronger among adult age groups, particularly those aged 18-24 and 25-31 years, while the trend among children aged 5-6 years was weaker than in the reference group.

## Discussion

To our knowledge, this is the largest nationwide population-based report examining the prescription of ADHD medication in Eastern European country.

The rise in the utilization of ADHD medication observed in our research aligns with global trends observed over the past few decades [4, 6, 9–12]. This trend could be explained by several factors, including increasing access to mental healthcare specialists (e.g. in Poland the number of child and adolescent psychiatrists increased from 296 in 2012 to 524 in 2023) [13], growing public awareness about ADHD, improvements in attitudes regarding pharmacological treatments, and the increase in the proportion of females recognized to have ADHD, as shown in our analysis. No new Polish guidelines changing the existing standards for ADHD diagnosis and treatment were published.

However, despite the observed increases, prescription rates in Poland remain significantly lower than expected when compared to studies from other countries. For instance, according to one analysis, the prescription rate in the United States between 2014 and 2019 ranged from 5.6 to 6.1 prescriptions per 100 persons. In the same period, based on our analyses, the corresponding rate in Poland was 0.24 to 0.33 prescriptions per 100 persons [14].

In another study, the prescription rate per 10,000 persons in the UK between 2012 and 2015 ranged from 295.9 (233.5, 374.9) to 394.4 (296.7, 524.2). During the same period, our analyses indicated that the corresponding rate in Poland was 17 to 22.7 prescriptions per 10,000 persons [15].

In several other publications, no discrepancies in ADHD prevalence across different regions of the world have been reported. Therefore, it is unlikely that the observed differences are due to lower ADHD prevalence in Poland compared to the USA and the UK [16–18].

Data from other European countries show similar patterns in ADHD medication use to those observed in our study. In Spain, ADHD medication was dispensed annually to approximately 1.77% of the population aged 0–19 years, with an increase of 48.9% over the study period (from 1.28% in 2010 to 1.9% in 2019), and the highest prevalence observed among children aged 10–14 years (3.42%). Methylphenidate was the most commonly used medication, in line with the predominance of this drug observed in our study [19]. In Germany, approximately 38% of patients diagnosed with ADHD received at least one prescription for ADHD medication, with a decrease observed among younger patients (≤ 16 years) and a substantial increase among adolescents and adults (e.g. a 113% increase in patients aged 17–24 years and a 355% increase among those ≥ 25 years) [20], which corresponds with our observation of a growing proportion of prescriptions in older age groups. In Slovenia, ADHD drug consumption increased from 0.0537 DDD/1000 inhabitants/day in 2001 to 0.3076 DDD/1000 inhabitants/day in 2012, with this growth being mainly driven by the increased use of methylphenidate - osmotic release oral delivery system and atomoxetine [21].

We believe that the lower number of prescriptions for ADHD medications in Poland may be attributed to several factors, such as an insufficient number of specialists in child and adolescent mental health and their uneven distribution across the country, public awareness that, despite growing, remains insufficient, the stigma associated with psychopharmacotherapy, and enduring social stereotypes surrounding its use.

Stable growth in the prescribing of ADHD medications was observed up to 2019, after which a noticeable acceleration occurred during the COVID-19 pandemic period – 2020–2022. This trend is likely due to increased ADHD symptoms and psychological difficulties caused by the social impacts resulting from COVID-19 such as lockdowns and remote working [22]. This observation is consistent with findings from other countries in Europe, with the exception of the absence of a decrease in 2020 [23]. This discrepancy could be attributed to differences in methodology. We relied on the number of prescriptions, so we do not have data on the exact quantity of medication dispensed at the individual level. Furthermore, since 2019, an increase in the proportion of adolescents and adults has been observed, along with a decrease in the relative proportion of preschoolers and children (Table 1). This shift in proportions between children and adolescents may be explained by the higher relative change values for female adolescents.

The increasing proportion of females and notable relative change in prevalence among adolescent and young adult females observed in our study may be partly explained by changes in DSM and ICD classifications. Firstly, DSM-IV and ICD-11 introduced the possibility of diagnosing the inattentive subtype of ADHD, which is particularly prevalent among females with ADHD [24]. Secondly, DSM-5 and ICD-11, by raising the age threshold for diagnosis, have made it possible to identify ADHD in a larger number of patients, including many females who were previously overlooked in diagnostic process. Females with ADHD may be more easily missed in the diagnostic process unless they have prominent externalizing problems, and consequently the diagnosis in females is typically later compared to males [25, 26].

The increase in prescription counts differed primarily by age group rather than sex. Adult age groups showed steeper temporal increases than children aged 7–12 years, suggesting that the overall increase was partly driven by a growing contribution of adolescent and adult prescriptions. However, these findings should be interpreted as heterogeneity in prescription counts rather than evidence of changes in the number of treated individuals.

Preschoolers exhibited distinct dynamics in prescription trends. After an initial period of increased prescription prevalence, a decline was observed, with indications of a resurgence in recent years (Fig. 2). The reasons for this pattern remain unclear. One possible explanation may be changes in diagnostic and treatment practices in younger children, including a more cautious approach to pharmacological treatment in this age group. Additionally, clinical guidelines typically recommend prioritizing non-pharmacological interventions in preschool-aged children, which may contribute to lower prescription rates. System-level factors, such as access to specialist care, may also play a role; however, the available data do not allow for a definitive explanation of this trend.

The age groups with the highest prevalence of ADHD medication usage were 7–12 and 13–17, similarly to results from retrospective observational study using population-based databases from multiple countries all over the world. Additionally, in the same study, the average relative increase was greater for adults than for youth, in line with our findings for the population of our study [4]. However, our study highlights a notably low percentage of adults compared to other age groups, indicating likely under-diagnosis of ADHD in adults in Poland. For example, a French study using a regional reimbursement database found that as early as 2011, the percentage of adults was 24.7% [27]. This comparison raises concerns not only about adherence to diagnostic guidelines and ADHD treatment in Polish adults but also about the transition from pediatric to adult mental healthcare systems.

The ratio of methylphenidate to atomoxetine prescriptions decreased substantially between 2012 and 2022, indicating a relatively faster increase in atomoxetine prescribing. The decreasing ratio of methylphenidate to atomoxetine observed in Poland, reflecting a relatively faster increase in atomoxetine prescribing, is not unique but appears to vary across European countries. In Slovenia, among children and adolescents, the number of patients treated with atomoxetine increased eight-fold between 2007 and 2015, while methylphenidate use increased by only 15%, indicating a stronger relative growth of second-line pharmacotherapy despite guideline recommendations [28]. In contrast, data from Scandinavian countries (Sweden, Norway, and Denmark) in paediatric and adolescent populations show more stable prescribing patterns, with slight decreases or minimal changes in methylphenidate and atomoxetine utilisation over time, alongside an increasing role of other ADHD medications [11]. Spanish data on children and adolescents similarly indicate an overall increase in ADHD medication use without a clear shift away from stimulant predominance [19].

However, it is difficult to draw definitive conclusions regarding the underlying causes of the observed pattern in Poland. Reimbursement policies are an unlikely explanation, as both methylphenidate and atomoxetine were introduced to reimbursement systems at a similar time. Current treatment guidelines recommend atomoxetine as a second-line option, to be used when stimulants are ineffective, poorly tolerated, or associated with a risk of misuse [29]. The observed prescribing pattern therefore raises questions regarding adherence to evidence-based ADHD treatment guidelines in clinical practice in Poland.

## Limitations

This study has certain limitations that should be acknowledged. Firstly, we relied solely on the available data on the number of prescriptions. This restricted our ability to provide data typically used in pharmacoepidemiological analyses, such as DDD (Defined Daily Dose), PDD (Prescribed Daily Dose), or the number of individuals taking a specific medication. This limitation complicates the comparison of medication usage with other countries.

Secondly, the lack of information on whether the prescriptions were actually dispensed means that our data primarily reflect prescription trends rather than actual drug utilization. Therefore, it is essential to conduct further research on the utilization of ADHD medications in Poland and Central and Eastern Europe. More accurate data would allow for the assessment of treatment persistence, age of treatment initiation, and drug utilization across different regions of Poland.

Thirdly, the database does not include information on patient comorbidities, which limits the ability to account for clinical complexity and potential treatment indications. Finally, no data on long-term treatment trajectories or persistence were available, which precludes evaluation of treatment duration, continuity, and long-term pharmacological patterns.

## Conclusion

An increase in the prescription rate may be a positive phenomenon, indicating that the number of individuals receiving help is growing, including those who were previously undiagnosed, such as women and young adults. However, the low prescription rates for ADHD medications in Poland compared to the UK and the US suggest that there is a significant group of individuals in Poland who have not been diagnosed. The number of prescriptions is likely to continue increasing in the coming years.

We consider our study to provide an important report on prescription trends in Poland, with particular relevance in the context of former East Bloc European countries. While underage individuals have received the majority of ADHD prescriptions, a growing number of adolescents and adults have also been prescribed ADHD medication. Despite this, adult prescribing rates in Poland remain low, suggesting underdiagnosis and limited transition support from pediatric to adult care. These findings underscore the need for improved ADHD diagnostic and treatment support, with particular focus on adults. Further research is also warranted using patient-based databases, which would allow for a more precise characterization of ADHD medication use in Poland.

## Data Availability

The data underpinning the findings of this study are not publicly disclosed. The competent authority responsible for their collection and custody is the Department of Health of the Ministry of Health of the Republic of Poland.
